# Ageism and the Pandemic: Risk and Protective Factors of Well-Being in Older People

**DOI:** 10.3390/geriatrics8010014

**Published:** 2023-01-16

**Authors:** Anna Rosa Donizzetti, Vincenza Capone

**Affiliations:** Department of Humanities, University of Naples Federico II, 80133 Naples, Italy

**Keywords:** well-being, ageism, COVID-19 age discrimination, loneliness, social isolation, positive affect, confidence in the future, COVID-19 outbreak

## Abstract

The COVID-19 pandemic has particularly affected the older population both in terms of the high number of victims and the psychological impact. Moreover, the pandemic has made older people more vulnerable to isolation and loneliness, and victims of ageism. The aim of the present study was to investigate the risk and protective factors for the well-being of older people during the pandemic. The role of positive affect, confidence in the future, current physical health, social isolation, loneliness, and ageism were analysed. A self-report questionnaire was administered to 1301 participants (mean age: 77.3 years, DS: 5.46), almost equally distributed by gender (56.1% female). Descriptive and correlational analyses were performed, together with SEM. The results showed that perceived age discrimination positively predicts loneliness and negatively and indirectly predicts well-being. Furthermore, positive affect, confidence in the future, and current physical health are protective factors, while loneliness, social isolation, and ageism are risk factors. Future emergency policies must take into account the impact of such actions on the well-being of this segment of the population.

## 1. Introduction

In late 2019 and early 2020, the ‘new coronavirus disease’ or COVID-19 [1] spread rapidly around the world, and as early as March 2020, the World Health Organisation was forced to declare pandemic status. The available data have shown that, in most people, the coronavirus produces more or less mild symptoms that recede within a few days. With increasing age, however, the risk of severe symptoms increases and it has been estimated that the mortality rate is ten times higher in the elderly over 60 than in adults under 60 [2]. The reason probably lies in the presence of at least one pre-existing disease condition among the elderly who died of COVID-19 [3,4]. Due to the rapid spread of the virus and the lack of adequate treatment to cope with it, many countries adopted containment measures from the outset, such as taking appropriate hygiene measures, wearing masks, physical removal, measuring temperature, suspending activities in cultural centres, etc. In addition to these measures, in the first phase of the spread of the virus, it was necessary to minimise contact between people, so much so that the Italian government, and others, decided to adopt a strict containment measure, i.e., the closure of all non-essential or strategic activities [5]. While social isolation served to reduce the spread of the virus, it also caused many psychological problems, such as increased stress, feelings of distress, anxiety, and depression [6,7]. These mental health problems have increased disproportionately especially among older people because during the pandemic they had reduced social networks, experienced greater feelings of loneliness, and had less access to social support [8,9,10], resulting in a negative impact on their quality of life [11].

Although the terms ‘social isolation’ and ‘loneliness’ are often used undifferentiated, they refer to different aspects [12] and, as numerous studies have shown, are also only weakly correlated [10,13,14]. Social isolation refers to the structure of the social network, which reflects the objective state of a lack of social relations [15,16]. Loneliness, on the other hand, is the feeling of a lack or loss of meaningful relationships; it is therefore a subjective phenomenon that reflects the quality of a person’s social interactions [17]. Therefore, loneliness occurs when there is a discrepancy between the quality and/or quantity of social relationships possessed by people [18]. Consequently, a person can feel lonely despite having a dense network of social relationships, just as, at the same time, he or she can be socially isolated and not feel lonely [19]. Social isolation and subjective feelings of loneliness could intensify reactions to any stress exposure such as the potential risk of infectious disease [10,20]. Furthermore, loneliness, being usually an unpleasant experience, is correlated with increased negative affect and decreased positive affect [21,22]. There are several risk factors associated with loneliness, such as: not being married/not having a partner and loss of a partner; a limited social network; a low level of participation in social activities; poor perceived health and depression/depressed mood [23]. Another risk factor, long neglected in the literature, is ageism [10,19]. The study by Donizzetti and Lagacé [10] highlighted not only that loneliness is the strongest antecedent of mental ill-health in older people, but also that it is a mediator of the effects of perceived age discrimination in the context of the COVID-19 pandemic on mental ill-health. During the most acute phases of the pandemic, a message that was constantly conveyed through the communiqués of government leaders and by the media in general was to advise older people to leave their homes only when necessary and to avoid frequenting crowded places [24]. Although these communications were aimed at protecting the frail, they often resulted in a paternalistic public communication that described all older people as ‘vulnerable’ members of society [25], a homogeneous segment of the population characterised by decline and uselessness, with a consequent reinforcement of intergenerational conflicts, prejudice, and discrimination [26].

The term ‘ageism’ refers to negative or positive stereotypes, prejudice, and/or discrimination against older people on the basis of their chronological age [27]. In Italy, it is males and young people who have higher levels of ageism than women and the older adult [28]. Moreover, poor knowledge of the ageing process, together with a high level of anxiety about ageing, represent the major antecedents of negative stereotypes towards older people, which, in turn, together with age, influence ageism [29]. Perceived age discrimination is generally decried as one of the main experiences of discrimination in a person’s life [30]. It is a subjective perception, as people’s behaviour can be interpreted differently depending on the situation [31]. Perceived age discrimination is also related to negative age stereotypes, as these can influence the behaviour of older people themselves, who perceive themselves as the object of such stereotypes. In fact, Levy’s Stereotype Embodiment Theory [32] highlights how stereotypes present in culture are internalised by people, resulting in self-definitions that affect functioning and health. Indeed, older people’s adherence to stereotypes about ageing acts as a self-fulfilling prophecy [33], significantly predicting negative effects on physical and mental functioning, sometimes even decades later [32]. Similarly, a positive self-perception of ageing is correlated with better functional health more than two decades later [34].

Perceiving that the measures taken during the pandemic were discriminatory towards them because they were linked to the generalisation of the frailty condition to all those over the age of 65 increased the feeling of loneliness, which was already widespread among older adults, and affected the feeling of fear and perception of the risk of contracting COVID-19 with direct and indirect effects on the malaise of the elderly [10]. However, one has to wonder what the risk and protective factors of mental health may have been. Indeed, from the perspective of the two continua model, mental illness and mental health are two related but distinct dimensions: one continuum indicates the presence or absence of mental health, the other the presence or absence of mental illness Keyes [35]. Positive mental health [35] is understood as the result of feelings of happiness and satisfaction with life (emotional well-being), positive individual functioning in terms of self-actualisation (psychological well-being), and positive social functioning in terms of being of social value (social well-being). Therefore, psychological health is not limited to the absence of psychological illness but includes the presence of a number of positive aspects such as positive affectivity and having a purpose in life [36,37,38].

Positive affectivity refers to the subjective experience of pleasant affective states, such as enthusiasm or joy, for shorter or longer intervals of time [39,40]. It affects the following aspects: physiological (better immune system: [41]), behavioural (more adaptive health behaviours, such as physical activity: [42]), and social (better social relationships: [43]). Furthermore, in a study of older people, positive affect was found to be associated with greater longevity [44]. These outcomes can be attributed to the tendency of people with high positive affect to positively evaluate ambiguous information about their own health, resulting in the adoption of better health practices. From this would also derive the ability of positive affect to produce indirect health effects [45]. Positive affect affects not only physical health but also mental health; this relationship has been observed in numerous studies [46,47]. Thus, it can be argued that the tendency to find positive meaning in general or adverse life situations helps to cope with them proactively, resulting in an increase in the initial level of positive affect [48]. Finally, another factor considered to be important for positive ageing, and thus for well-being, is confidence in the future, understood as confidence in one’s present condition but situated in future time [49]. Confidence in the future, which points to a brighter anticipated future, has the potential to realise positive outcomes. Future confidence could presumably provide cognitive power in the process of adapting to an emergency situation such as a pandemic.

### The Current Research

The aim of the present study was to investigate the positive mental health of older people during the COVID-19 pandemic. To this end, we want to investigate the role that various factors may have on the mental well-being of older people, such as positive affect, confidence in the future, current physical health (as positive factors), social isolation, loneliness, and perceived age discrimination in the context of the COVID-19 pandemic (as negative factors).

To this end, the following assumptions were made:

**Hypothesis 1.**  *We expected that well-being was positively correlated with measures of positive affect, confidence in the future, and physical health (H1a); while it was negatively correlated with measures of perceived age discrimination in the context of the COVID-19 pandemic, loneliness, and social isolation (H1b).*

**Figure 1:**
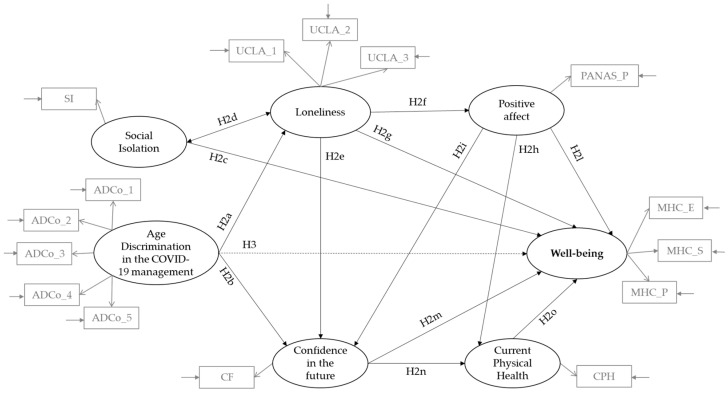
Conceptual Model.

**Hypothesis 2.**  *Based on the literature review, we constructed an a priori model to be tested. As shown in Figure 1* *, we expected perceived age discrimination in the context of the COVID-19 pandemic to be a positive antecedent of loneliness (H2a) and a negative antecedent of confidence in the future (H2b); that social isolation was a negative antecedent of well-being (H2c) and was positively correlated with loneliness (H2d); that loneliness was a negative antecedent of confidence in the future (H2e), positive affectivity (H2f) and well-being (H2g); that positive affectivity was a positive antecedent of current physical health (H2h), confidence in the future (H2i), and well-being (H2l); finally, we expected confidence in the future to be a positive antecedent of well-being (H2m) and physical health (H2n), which in turn affected well-being (H2o).*

**Hypothesis 3.**  *Finally, we expected an indirect effect of perceived age discrimination during the COVID-19 pandemic on well-being, with a mediating effect of loneliness (H3).*

## 2. Materials and Methods

### 2.1. Procedure of Recruitment and Participants

The study, which complied with the ethical principles of the 1995 Declaration of Helsinki and was conducted in accordance with APA ethical standards, was approved by the local ethics committee of the principal investigator’s institution (protocol code: 6/2021 of 19 February 2021).

Participants were contacted in May and June 2021 through non-probabilistic snowball sampling. In particular, a group of university students was asked to administer the questionnaire to two older acquaintances, who in turn provided the contact of other older adults and, through word of mouth, other participants were reached. The only inclusion criterion was age over 65.

Participants were asked to answer an online questionnaire on their perceptions during the COVID-19 pandemic. They were invited to complete the questionnaire via a web link associated with the Google Forms platform. Participation in the study was voluntary and anonymous, and participants could leave the study at any time. The average time for completion was approximately 30 min.

Informed consent was asked on the first page of the survey, then the form included different scales to measure: loneliness, well-being, age discrimination in coping with COVID-19, social isolation index, positive affectivity, hope for the future and physical health status (for a detailed description of the instruments, see the following section). The survey also included a short demographic section to collect information on participants’ age, gender, educational level, marital status, and education level, as well as a series of questions on the extent and frequency of their social network and participation in recreational activities.

The 1301 Italian participants were almost equally distributed by gender (56.1% female, 43.9% male) and were aged between 67 and 99 years (M = 77.25, SD = 5.46). Most of the participants reside in southern Italy (93.5%), while the remainder are distributed between central (4.5%) and northern Italy (2.0%). Only two people claim not to be Italian citizens. The level of education was quite low, with 49.80% having a primary school qualification, 25.00% a junior high school qualification, and 18.70% a high school qualification; a small percentage had a university degree (5.70%) or postgraduate degree (0.80%). Over half of the participants stated that they were married or cohabiting (57.10%), while the remainder were widower (38.20%), single (2.5%) or divorced/separated (2.20%). With regard to living situation, 74.90% lived with at least one family member (M = 2.83; SD = 1.31) and 25.10% of the participants lived alone.

### 2.2. Instruments

The measures used in the survey are described below:

Mental Health Continuum-Short Form (MHC-SF) [50,51]. The MHC-SF consists of 14 items on 6-point scales, ranging from 0 = never to 5 = every day. It measures the degree of emotional well-being (e.g., of item: “During the past month, how often did you feel happy”; α = 0.82), social well-being (e.g., of item: “During the past month, how often did you feel that you belonged to a community”; α = 0.77), and psychological well-being (e.g., of item: “During the past month, how often did you feel that you had warm and trusting relationships with others”; α = 0.84). The internal reliability was 0.89 in this study.

Age discrimination scale in the context of the COVID-19 pandemic (ADCo). Perceived age discrimination about the management of COVID-19 was measured using five items assessed on a Likert scale from 1 (strongly disagree) to 5 (strongly agree). The items were adapted from Garstka’s scale [52] and are as follows: “I feel I am victim of the government’s Coronavirus policies because of my age”; “In this pandemic period, members of my age group have been discriminated against more than members of other age groups”; “During this pandemic period, members of my age group did not receive the same care as members of other age groups because of their age”; “In this pandemic period I feel that I have been deprived of the opportunities for others because of my age”; “I feel that social media (news, newspapers, etc.) has discriminated against me and members of my group because of the way the pandemic and its effects have been presented”. The mean of these five items was calculated, with higher values representing greater perceived discrimination. In the current study, the Cronbach’s α of the scale is 0.83.

UCLA Loneliness Scale-version 3 (UCLA) [53,54]. The UCLA Loneliness Scale consists of 20 items for the global measurement of loneliness (e.g., “I feel isolated from others”). The items are evaluated on a four-point Likert scale, ranging from 1 (never) to 4 (always). Nine items are positively formulated and reversed. The scale consists of three dimensions (Isolation, Relational Connectedness, and ‘Trait’ Loneliness), but for this study, only the overall measure was considered. In this study, the internal consistency reliability of the scale is 0.88 (Cronbach’s α).

Social Isolation Index (SII). In line with the work of Wister et al. [55], to obtain a measure of social isolation, an index was calculated by averaging the following variables: social network quantity, attendance in presence in the last month, attendance through technology in the last month, and community participation (see [10]). The scores range from 0 = every day to 5 = never; therefore, high values correspond to a higher index of social isolation.

The Positive Affect Schedule (PANAS_P) [56,57] reflects the extent to which a person feels enthusiastic, active, and alert. It comprises five descriptors of positive affect, rated for their momentary presence on a 7-point Likert-type scale, ranging from 1 (not at all) to 7 (extremely). Cronbach’s alpha for PA was 0.84.

Confidence in the Future (CF). Confidence in the future was measured through the item “Did you have hope and confidence in the future” taken from the CES-D scale [58,59], which measured symptoms of depression in community populations. Subjects are asked to rate each item on a scale from 1 (rarely or none of the time; less than 1 day), to 4 (most or all of the time; 5–7 days).

Current Physical Health (CPH). Current physical health was measured by the item “Thinking about the last month, how do you rate your health condition?”, rated on a 5-point Likert scale (1 = very bad to 5 = very good).

### 2.3. Statistical Analysis

The survey data were entered into SPSS 22.0 databases and M-Plus 6.1 software. Cronbach’s alpha was used to calculate the reliability of the scales. For the psychological scale, an internal consistency should be greater than 0.70 (however, an alpha between 0.60 and 0.69 is considered acceptable [60]).

Descriptive statistics (mean, standard deviation, skewness, and kurtosis) were first performed on data. Pearson’s correlation coefficients were also calculated to determine associations between variables under study. Second, Structural Equation Modelling (SEM) [61] was conducted to test the hypothesised model. To assess goodness-of-fit of that model, the following indicators were used: Chi-squared distribution and the degrees of freedom (χ^2^/df ≤ 3), although this index is sensitive to sample size; Standardised Root Mean Square Residual (SRMR ≤ 0.09); Comparative Fit Index (CFI > 0.90); and Tucker–Lewis index (TLI > 0.90). If the results of the Root Mean Square Error of Approximation (RMSEA) are ≤0.05, they are considered to be good, and reasonable if they are ≤0.08, with a cut-off value of 0.06. In order to assess the goodness of the model, several indices will be considered simultaneously as the different indices assess different aspects of the goodness-of-fit [62,63,64,65]. Satisfactory models should show consistently good-fitting results on many different indices.

## 3. Results

### 3.1. Descriptive Statistics and Correlation Analysis

Means, standard deviations, skewness, kurtosis, and correlations are shown in Table 1.

As shown in Table 1, well-being is positively correlated with measures of positive affect (0.67 **), confidence in the future (0.45 **), and physical health (0.35 **), while it is negatively correlated with measures of loneliness (−0.56 **), social isolation (−0.42 **), and perceived age discrimination in the context of the COVID-19 pandemic (−0.16 **).

### 3.2. Testing of the Hypothesised Conceptual Model

**Figure 2:**
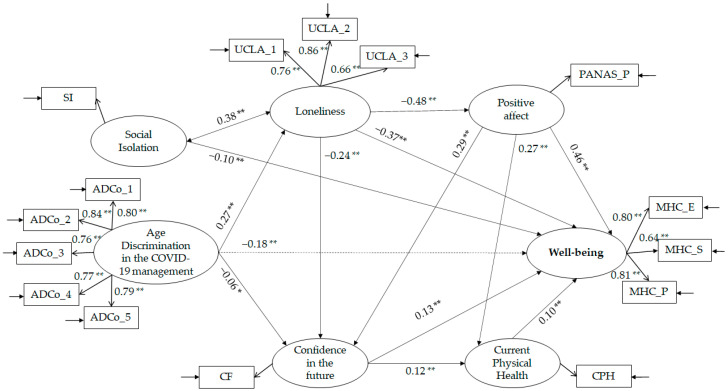
Structural equation models with standardised coefficient estimates (** *p* < 0.001; * *p* = 0.05).

We used structural equation modelling to test the hypothesised relationships between variables under study (see Figure 2). The results suggest an acceptable fit between the theoretical and empirical models: χ^2^ (df) = 394.040 (80), *p* ≤ 0.001; χ^2^/df = 4.925; CFI = 0.963; TLI = 0.952; RMSEA = 0.055 (0.050–0.060); and SRMR = 0.039. All the hypothesised relationships were significant. Precisely, as hypothesised, perceived age discrimination in the context of the COVID-19 pandemic positively predicts loneliness (H2a; β = 0.27 **) and confidence in the future (H2b; β = −0.06 *). Social isolation is a negative predictor of well-being (H2c; β = −0.10 **) and is positively correlated with loneliness (H2d; β = 0.38 **). Loneliness negatively predicts confidence in the future (H3e; β = −0.24 **), positive affect (H3f; β = −0.48 **), and well-being (H3g; β = −0.37 **). Positive affect is a positive antecedent of current physical health (H3h; β = 0.27 **), confidence in the future (H3i; β = 0.29 **), and predicts well-being (H3l; β = 0.46 **). Confidence in the future is an antecedent of well-being (H3m; β = 0.13 **) and current physical health (H3n; β = 0.12 **), which in turn predicts well-being (H3o; β = 0.10 **).

While perceived age discrimination in the context of the COVID-19 pandemic does not directly affect well-being (dashed lines in Figure 2), an indirect effect emerged. Specifically, perceived age discrimination in the context of the COVID-19 pandemic affects well-being through the mediation of loneliness (H3; β = −0.18 **).

## 4. Discussion

Today’s society is faced with two major challenges: an ageing population and the management of a global pandemic. The demographic revolution, which is affecting industrialised countries in particular, has led scholars to turn their attention to the bio-psycho-social factors involved in the ageing process, in order to promote and support the development of those factors that underpin positive ageing. The scientific acquisitions obtained in recent decades must, however, be reread in light of the worldwide pandemic that has completely disrupted lifestyles, exposing the entire population, and especially older people, to continuous sources of stress that have transformed their bio-psycho-social balance. The aim of this study was to investigate which factors can compromise the mental health of older people and which, moreover, can act as protective factors from the perspective of Keyes’ two continua model [35], which considers mental illness and mental health as two distinct but related dimensions. Risk factors included loneliness, social isolation, and perceived age discrimination in the context of the COVID-19 pandemic; protective factors included positive affect, confidence in the future, and good health.

The hypotheses formulated were all confirmed. The analyses conducted showed that, in line with the first hypothesis, well-being is negatively correlated with loneliness, social isolation, and perceived age discrimination in the context of the COVID-19 pandemic [11,66,67], while it is positively correlated with positive affect, confidence in the future, and physical health [68,69]. With regard to the second hypothesis, the model tested presents good fit indices and the relationships were all confirmed. The model shows that the strongest positive antecedent of well-being is positive affect, confirming the latter’s link with mental health [46,47]. Furthermore, positive affect is a positive antecedent of both future confidence and physical health [48]. This finding confirms that people with higher positive affect are able to have a positive outlook on the future even in an emergency situation and are able to be proactive in implementing health practices to preserve their health status, even during a pandemic. Confidence in the future and physical health are also correlated with mental well-being, albeit with less predictive power than positive affect. Positive affect, however, suffers from the negative effect of loneliness, as it is an unpleasant experience [21,22]. Loneliness in turn correlates with social isolation [17] and is predicted by perceived age discrimination in the context of the COVID-19 pandemic. Each of these dimensions negatively affects well-being, albeit with different predictive power. Furthermore, for well-being as well as for malaise [10], perceived age discrimination in the context of the COVID-19 pandemic does not affect people in a direct way, but through the mediation of loneliness (third hypothesis). The latter negatively affects confidence in the future, probably because people who feel they have been treated unfairly because of their age tend to project this experience into the future. Despite the fact that ageism is a widely experienced form of prejudice and discrimination, experiences of ageism remain relatively unstudied [70], as it is a widely tolerated form of prejudice and is considered an inevitable consequence of the ageing process [71,72]. Since a crisis can also be seen as an opportunity for change, the COVID-19 pandemic could be an opportunity to make it clear how less favourably older people are treated because of their age. The results of this work highlight a number of protective factors that can be implemented not only by individual communities, but also by government institutions. It is clear that although the pandemic has changed its face and we are no longer in an emergency situation, older people remain the most affected by this crisis. This is why action plans to improve well-being and reduce ill health are essential. It is, therefore, confirmed in our study that the self-reported experience of age discrimination is negatively associated with well-being, as a few other studies had also previously shown [73,74]. In future studies it will be important to investigate ageism and how negative attitudes towards age can affect the potential for healthy and active ageing [72], such as physical functioning, health, and quality of life, in line with what the World Health Organisation (WHO) advocates.

### 4.1. Limitations

This study has limitations, which will have to be overcome in future studies. Firstly, it is a cross-sectional study and this prevented the examination of causal relationships over time. Therefore, the relationships examined should be investigated in future longitudinal investigations that also take into account the temporal dimension, in order to grasp the causal links between them. Second, it is a single-method study, as only self-report instruments were used, and this may have led to the inflation of an observed association. In this sense, future surveys could envisage the integration of qualitative study methods that would make it possible to capture aspects that self-report instruments are unable to detect, especially in view of the type of population being addressed. Thirdly, the snowball sample, besides not being representative of the population, may have led to finding an overly homogeneous group of subjects, also in view of the concentration of the sample in southern Italy. Therefore, the sample needs to be enlarged by considering other types of recruitment that would make it possible to contact people from different geographical areas and be more diversified in terms of education and social background.

### 4.2. Implications

This study considered several factors involved in the ageing process and the pandemic, making it possible to simultaneously analyse existing relationships and provide an overview of the constructs analysed. From the data that emerged, it was possible to capture the role of protective and risk factors in the well-being of older people; among these, the role of loneliness and positive affectivity emerged, as well as that of the perception of having suffered age discrimination in the way the pandemic was handled. The latter exacerbated feelings of loneliness and reduced expectations of the future with repercussions on levels of well-being. Why did older people feel discriminated against? Firstly, the call for all older people to reduce social contact was perceived as unfair because it was a generalisation of frailty to all those over 65 years of age, who are not necessarily all in a more vulnerable condition [75]. Secondly, older people felt discriminated against because they perceived that they did not have the same opportunities for care as younger people, especially in the most acute phase of the pandemic when, due to the shortage of healthcare resources, it was necessary to choose who to care for. Finally, we must take into account the messages conveyed by social media, which are responsible for presenting the COVID-19 pandemic as an ‘older adult problem’ by altering personal perceptions of susceptibility to COVID-19 and influencing the health behaviour of young and old. In the light of these important elements, we can draw useful pointers for decision-makers who, in the future, must take these elements into account not only in the management of emergency situations but also and above all in everyday dynamics. It is necessary to make more conscious use of communication and to plan interventions aimed at preventing loneliness, including through the enhancement of positive affectivity, and social skills, the improvement of social support, and the expansion of opportunities for social interaction [76]. One strategy could be giving older people a voice in order to promote their inclusiveness [77] and to make the diversity that exists in this age group evident in the eyes of other generations. Combating ageism requires a concerted effort by all stakeholders to convey positive messages associated with ageing and create an environment of respect, empathy, and solidarity towards older people, especially during the COVID-19 pandemic [77].

## Figures and Tables

**Table 1:** Means, standard deviations, skewness, kurtosis, and correlations between the variables included in the study.

	Means (SD)	Range	Skew	Kurt	1	2	3	4	5	6	7
1. Well-being (MHC-SF)	2.64 (1.00)	0–5	−0.04	−0.47	1						
2. Age Discrimination in the Context of the COVID-19 Pandemic (ADCo)	2.75 (0.96)	1–5	0.28	−0.50	−0.16 **	1					
3. Loneliness (UCLA)	2.10 (0.47)	1–4	0.24	−0.19	−0.56 **	0.24 **	1				
4. Social Isolation (SII)	2.92 (0.64)	0–5	0.04	−0.32	−0.42 **	0.03	0.32 **	1			
5. Positive Affect (PA)	2.57 (0.91)	1–5	0.24	−0.43	0.67 **	−0.13 **	−0.42 **	−0.36 **	1		
6. Confidence in the Future (CF)	2.84 (0.88)	1–4	−0.26	−0.73	0.45 **	−0.15 **	−0.35 **	−0.22 **	0.41 **	1	
7. Current Physical Health (CPH)	3.24 (0.83)	1–5	−0.37	0.20	0.35 **	−0.12 **	−0.28 **	−0.20 **	0.32 **	0.23 **	1

Notes: ** *p* < 0.01.

## Data Availability

All study data are included in the present manuscript.
